# Carbapenem-Resistant *Enterobacteriaceae* Infections: Results From a Retrospective Series and Implications for the Design of Prospective Clinical Trials

**DOI:** 10.1093/ofid/ofx063

**Published:** 2017-06-01

**Authors:** Elizabeth L. Alexander, Jeffery Loutit, Mario Tumbarello, Richard Wunderink, Tim Felton, George Daikos, Karen Fusaro, Dan White, Shu Zhang, Michael N. Dudley

**Affiliations:** 1 Infectious Diseases Care Global Innovation Group, The Medicines Company, Parsippany, New Jersey; 2 Infectious Diseases Care Global Innovation Group, Rempex Pharmaceuticals (a wholly-owned subsidiary of The Medicines Company), San Diego, California; 3 Institute of Infectious Diseases, Catholic University of the Sacred Heart, A. Gemelli Hospital, Rome, Italy; 4 Pulmonary and Critical Care Medicine, Northwestern University-Feinberg School of Medicine, Chicago, Illinois; 5 Centre for Respiratory Medicine and Allergy, University of Manchester, United Kingdom; and; 6 First Department of Propedeutic Medicine, University of Athens, Medical School, Greece

**Keywords:** antibiotics, carbapenem-resistant *Enterobacteriaceae*, clinical trials, mortality

## Abstract

**Background:**

The increasing incidence of multidrug-resistant Gram negatives, such as carbapenem-resistant *Enterobacteriaceae* (CRE), has resulted in a critical need for new antimicrobials. Most studies of new antimicrobials have been performed in patients with nondrug-resistant pathogens. We performed a retrospective analysis of patients with CRE infections to inform the design of phase 3 clinical trials.

**Methods:**

This was a retrospective study at 22 centers in 4 countries. Baseline data, treatment, and outcomes were collected in patients with complicated urinary tract infection (cUTI)/acute pyelonephritis (AP), hospital-acquired bacterial pneumonia (HABP), ventilator-associated bacterial pneumonia (VABP), and bacteremia due to CRE.

**Results:**

Two hundred fifty-six cases of CRE infection were identified: 75 cUTI/AP, 21 HABP, 20 VABP, and 140 bacteremia. The patient population had significant comorbidities: 32.8% had chronic renal insufficiency, and 26.2% were immunocompromised. Illness severity at presentation was high: 29.3% presented with septic shock. Treatment regimens varied widely; however, a majority of patients received combination therapy. Outcomes were universally poor (28-day mortality was 28.1%) across all sites of infection, particularly in dialysis patients and those with sepsis.

**Conclusions:**

The CRE infections occured in patients with substantial comorbidities and were associated with high mortality and low rates of clinical cure with available antibiotics. Patients with these comorbidities are often excluded from enrollment in clinical trials for registration of new drugs. These results led to changes in the inclusion/exclusion criteria of a phase 3 trial to better represent the patient population with CRE infections and enable enrollment. Observational studies may become increasingly important to guide clinical trial design, inform on the existing standard of care, and provide an external control for subsequent trials.

The increasing incidence of drug-resistant infections has resulted in a critical need for new antimicrobial agents [1, 2]. As noted by regulators, statisticians, and clinical investigators, conducting clinical trials in patients with drug-resistant infections presents considerable challenges [3–5]. Thus, most studies of new antibacterial agents have been limited to comparative studies in patients with infections due to pathogens susceptible to approved antimicrobial agents [6]. As an alternative strategy, new agents may be studied in patients with multidrug-resistant infections, and compared against “best available therapy”, where therapy is individualized based on local surveillance data and adjusted based on in vitro susceptibility of cultured isolates [7, 8].

The new agent meropenem-vaborbactam combines meropenem with vaborbactam, a first-in-class β-lactamase inhibitor that restores meropenem activity against carbapenem-resistant *Enterobacteriaceae* (CRE) producing the *Klebsiella pneumoniae* carbapenemase (KPC) [9]. As part of the development of meropenem-vaborbactam, a prospective, randomized phase 3 trial of meropenem-vaborbactam versus best available therapy in patients with infections due to CRE is ongoing (Clinical Trial NCT02168946) [7]. In designing this study, we recognized that patients with CRE infections may not be typical of patients enrolled in standard antimicrobial clinical trials [10–12]. Therefore, we conducted a retrospective analysis of patient characteristics, antimicrobial treatments, and outcomes associated with CRE infections at hospitals that would participate as study sites. The purpose of this retrospective analysis was to inform on the final protocol design of the prospective phase 3 trial, in particular the inclusion and exclusion criteria [7].

## MATERIALS AND METHODS

### Objectives

The primary objective of this observational analysis was to describe the patient characteristics, comorbidities, treatment, and outcomes of serious infections due to CRE with currently available therapy. The secondary objective was to inform the design of a phase 3 study [7] to determine whether (1) regulatory-based disease definitions apply to patients with CRE infections, (2) standard clinical trial exclusion criteria apply to patients with CRE infections, and (3) there exists a single “best alternative treatment” (standard of care) for CRE infections.

### Setting, Study Design, and Participants

This was a retrospective analysis of patients with CRE infection at 22 major medical centers in 4 countries (United States, United Kingdom, Italy, and Greece) over 6 months. Sites were chosen based on CRE incidence and predominance of KPC production as the mechanism of carbapenem resistance at that site. Patients were included if they were adults with CRE pathogens identified from urine, blood, or respiratory samples submitted in the setting of a corresponding complicated urinary tract infection (cUTI) or acute pyelonephritis (AP), hospital-acquired bacterial pneumonia (HABP), ventilator-associated bacterial pneumonia (VABP), or bacteremia that occurred between September 1, 2013 and March 1, 2014. Any patient who, in the opinion of the treating physician, had clinical evidence of cUTI/AP, HABP, VABP, or bacteremia requiring antimicrobial treatment was eligible for inclusion. Investigators were encouraged to submit all qualifying consecutive cases. Recurrent infections (ie, same patient) were excluded. In cases of concurrent infection, patients were classified according to the infection deemed by the investigator to be the primary CRE infection. Patients’ data were compiled until death or hospital discharge.

### Definitions

#### Clinical Characteristics

A prior culture positive for CRE was defined as a culture for CRE obtained from any site (including surveillance cultures) within 90 days before the qualifying CRE infection. Immunocompromised conditions included hematologic malignancy, prior bone marrow transplant, or receipt of immunosuppressive therapy, antirejection medications for transplantation, or long-term (>2 weeks) use of systemic steroids (regardless of dose). Severe sepsis was defined as infection associated with any of the following: hypotension (systolic blood pressure [SBP] ≤90 mmHg or a decrease in SBP of ≥40 mmHg from baseline unresponsive to fluid challenge), hypothermia (core temperature <35.6°C or <96.1°F), or disseminated intravascular coagulation (DIC) as evidenced by prothrombin time or partial thromboplastin time 2× the upper limit of normal or platelets less than 50% of the lower limit of normal [13]. Septic shock was defined as infection associated with hypotension [13]. Fever was defined as oral or tympanic temperature ≥38°C or rectal temperature of ≥38.3°C. Leukocytosis was defined as white blood cell count (WBC) ≥10 000 cells/mm^3^, and “bandemia” was defined as ≥15% immature polymorphonuclear cells regardless of the total WBC count. Pyuria was defined by either a positive leukocyte esterase on urinalysis, presence of WBC >10 cells/mm^3^ in unspun urine, or WBC >10 cells/high-power field in sediment. Clinical cure was defined as resolution of signs and symptoms, as ascertained by the study investigator, such that no further antibiotics for the treatment of the index CRE infection were needed.

#### Microbiologic Definitions

Each hospital conducted susceptibility testing according to its own protocol. For all antimicrobial agents other than carbapenems, isolates were considered “susceptible” to a given antimicrobial agent based on the report by the local microbiology laboratory. Isolates were considered “non-susceptible” when results of local testing were recorded as “intermediate” or “resistant”.

Carbapenem resistance was defined according to revised criteria provided in 2014 Clinical and Laboratory Standards Institute (CLSI) breakpoint definitions. These definitions were provided to the sites, and sites were requested to report results of carbapenem susceptibility testing accordingly (CLSI M100-S24, page 55) [14]. “Index CRE isolate” was defined as the first isolate entered by the site in the electronic case report form (eCRF) under “qualifying CRE organism” that was a member of the family *Enterobacteriaceae* and met this definition of carbapenem resistance.

#### Treatment Definitions

Empiric treatment was defined as any antimicrobial treatment that began before the date on which all susceptibility data were available for the index CRE pathogen. Directed treatment was defined as any antimicrobial treatment that began on or after the date on which all susceptibility data were available for the index CRE pathogen. Both were further categorized as monotherapy or combination therapy according to the total number of antimicrobial agents with activity against aerobic Gram-negative bacteria used in combination for at least 3 calendar days. Empiric and directed therapies were also categorized according to the number of agents with in vitro activity against the index CRE pathogen used together for at least 3 calendar days. The in vitro activity of each agent against the index CRE pathogen was determined according to the microbiological data entered in the eCRF.

Microbiological eradication was defined as absence of the index CRE pathogen on follow-up cultures. Minimum inhibitory concentration testing was performed on each isolate by the clinical microbiology laboratory of the local site, according to procedures used by the local microbiology laboratory and captured in the database. If available, molecular testing for mechanism of carbapenem resistance was recorded.

### Data Collection and Analysis

Medical records were reviewed to identify patient demographics, CRE risk factors, comorbidities, site of infection, infection characteristics, microbiology (of both the index CRE pathogen and other pathogens), antimicrobial treatments, and outcomes. Risk factors associated with CRE infection were examined, including prior positive culture for CRE, prolonged hospitalization, immunocompromised condition, neutropenia, prior transplantation, and site-specific risk factors (eg, indwelling invasive devices at index culture; neurogenic bladder with urine residual volume ≥100 mL [for cUTI]) [11, 12, 15–16]. Presence or absence of baseline risk factors, symptoms, laboratory findings, and physical exam findings characteristic of cUTI/AP, HABP, VABP, and bacteremia according to regulatory guidance-based definitions [17–19] were collected to inform on the extent to which those characteristics reliably apply to patients with CRE infections. Additional patient variables collected included comorbidities [20], presence of concurrent bacteremia, presence of severe sepsis or septic shock at initial presentation with index CRE infection, and Acute Physiologic Assessment and Chronic Health Evaluation (APACHE) II score at the time of presentation with index CRE infection [21].

The primary outcome measure was 28-day mortality. Subjects in whom mortality data were not available were censored. Secondary outcomes included clinical cure (defined above), microbiologic eradication, duration of hospitalization for treatment of the index CRE infection, and duration of intensive care unit stay (if any) for index CRE infection.

### Statistical Analyses

Data were tabulated and descriptive summary statistics were conducted. Continuous endpoints were analyzed through a mixed-effects model, and categorical endpoints were analyzed by logistic regression. To determine the effect of combination therapy (either empiric or directed) on clinical outcome and 28-day mortality rates, 2 definitions were used. In the first, combination therapy was defined as 2 or more agents with Gram-negative therapy used in combination (≥3 days). In the second, combination therapy was defined as 2 or more agents with in vitro activity against the index CRE pathogen used in combination (≥3 days). For both empiric and directed therapies and both definitions, odds ratios were calculated to compare combination therapy versus monotherapy for study outcomes of clinical cure and 28-day survival (Supplemental Table I).

For the multivariate analysis of factors associated with 28-day mortality across all infection types, 27 patients with missing 28-day mortality data were excluded from the analysis. Multivariate logistic analysis was then performed using the initial covariates shown in Supplemental Table F. The final model was achieved using stepwise selection with entry and removal significance levels of 0.10, and the result is presented in Supplemental Table E.

## RESULTS

### Patient Characteristics and Infection Sites

Two hundred fifty-six cases of CRE infections (75 cUTI/AP, 21 HABP, 20 VABP, and 140 cases of bacteremia) were identified. The average age of patients was 62 years with a male predominance across all disease types. Ninety-nine patients (38.7%) had a prior positive culture for CRE within the preceding 90 days.

Bacteremia and cUTI/AP were the most frequent infection types reported and comprised 54.7% (140 of 256) and 29.3% (75 of 256) of the study population, respectively. Among the 68 patients with cUTI, a majority had a previous indwelling urinary catheter (47 of 68, 69.1%). The most common symptoms, signs, or laboratory results in patients with cUTI and AP were fever (35 of 75, 46.7%), leukocytosis/bandemia (44 of 75, 58.7%), and pyuria (61 of 75, 81.3%). Of the 21 patients enrolled with HABP, the most common characteristics were new-onset need for mechanical ventilation (12 of 21 patients, 57.1%), fever (13 of 21, 61.9%), and leukocytosis (10 of 21, 47.6%). The most common respiratory findings in patients with VABP were worsening partial pressure oxygen/fraction-inspired oxygen (PaO_2_/FiO_2_) ratio (15 of 20, 75%), auscultatory findings consistent with pneumonia (15 of 20, 75%), and need for increased suctioning (16 of 20, 80%). In addition, 14 of 20 (70%) patients with VABP had fevers or hypothermia (rectal or core temperature <35°C), and 16 of 20 (80%) had leukocytosis. Among patients with CRE bacteremia, the most common known source was intravenous catheter in 47 of 140 (33.6%) patients. The most common signs and symptoms were fever (94 of 140, 67.1%), leukocytosis (84 of 140, 60%), and tachycardia (94 of 140, 67.1%). It is worth noting that among the 68 patients with cUTI, 16 (23.5%) had concurrent CRE bacteremia. This was similar across other indications; 5 of 21 (23.8%) patients with HABP and 5 of 20 (25%) patients with VABP had concurrent bacteremia (Table 1).

**Table 1.** Characteristics of the Study Cohort Stratified by Site of Infection

Variable	Number (%) of Patients				
	Infection Type				
	Bacteremia^a^ (*N* = 140)	HABP (*N* = 21)	VABP (*N* = 20)	cUTI/AP (*N* = 75)	All (*N* = 256)
Demographic Variables					
Male sex	87 (62.1%)	15 (71.4%)	13 (65.0%)	35 (46.7%)	150 (58.6%)
Age, years, mean (SD)	62.3 (15.6)	60.3 (16.9)	55.5 (17.2)	62.7 (16.8)	61.7 (16.2)
Country of Residence					
** ** Greece	11 (7.9%)	0	4 (20.0%)	0	15 (5.9%)
** **Italy	67 (47.9%)	9 (42.9%)	6 (30.0%)	12 (16.0%)	94 (36.7%)
** ** United Kingdom	12 (8.6%)	1 (4.8%)	0	2 (2.7%)	15 (5.9%)
** ** United States	50 (35.7%)	11 (52.4%)	10 (50.0%)	61 (81.3%)	132 (51.6%)
Patient-Specific Risk Factors					
Prior culture positive for CRE	74 (52.9%)	3 (14.3%)	7 (35.0%)	15 (20.0%)	99 (38.7%)
Duration of hospitalization, mean (SD)^b,j^	27.5 (40.1)	22.7 (24.6)	17.5 (22.6)	13.2 (20.2)	22.2 (33.5)
Immunocompromised condition^c^	36 (25.7%)	7 (33.3%)	6 (30.0%)	18 (24.6%)	67 (26.2%)
Presence of neutropenia^d^	14 (10.0%)	0	1 (5.0%)	2 (2.7%)	17 (6.6%)
Prior transplantation^e^	24 (17.1%)	3 (14.3%)	4 (20.0%)	10 (13.3%)	41 (16.0%)
** ** Renal	5 (3.6%)	1 (4.8%)	0	2 (2.7%)	8 (3.1%)
** ** Hepatic	6 (4.3%)	2 (9.5%)	2 (10.0%)	5 (6.7%)	15 (5.9%)
Heart/lung	2 (1.4%)	0	0	0	2 (0.8%)
Other	13 (9.3%)	0	2 (10.0%)	4 (5.3%)	19 (7.4%)
Comorbidities					
Diabetes mellitus	41 (29.3%)	4 (19.0%)	5 (25.0%)	22 (29.3%)	72 (28.1%)
Heart failure^j^	26 (18.6%)	6 (28.6%)	6 (30.0%)	16 (21.3%)	54 (21.1%)
Chronic renal insufficiency^f^	47 (33.6%)	5 (23.8%)	5 (25.0%)	27 (36.0%)	84 (32.8%)
Requirement for dialysis	32 (22.9%)	2 (9.5%)	9 (45.0%)	8 (10.7%)	51 (19.9%)
Solid tumor	34 (24.3%)	7 (33.3%)	3 (15.0%)	12 (16.0%)	56 (21.9%)
Hematologic malignancy	22 (15.7%)	3 (14.3%)	1 (5.0%)	8 (10.7%)	34 (13.3%)
Concurrent bacteremia	NA*	5 (23.8%)	5 (25%)	16 (23.5%)	NA
Charlson Comorbidity Index, median (IQR)	3.0 (2.0–5.0)	4.0 (2.0–7.0)	3.0 (3.0–4.5)	3.0 (2.0–6.0)	3.0 (2.0–5.0)
Empiric therapy without in vitro activity^g^	80 (57.1%)	17 (81%)	16 (80%)	60 (80.0%)	173 (67.6%)
Presentation with severe sepsis^h,j^	52 (37.1%)	6 (28.6%)	11 (55.0%)	15 (20.0%)	84 (32.8%)
Presentation with septic shock^i^	47 (33.6%)	6 (28.6%)	8 (40.0%)	14 (18.7%)	75 (29.3%)
APACHE II score, mean (SD)	22.1 (10.5)	18.6 (9.5)	21.4 (6.3)	23.5 (9.0)	21.9 (9.7)

Abbreviations: AP, acute pyelonephritis; APACHE, Acute Physiologic Assessment and Chronic Health Evaluation; CI, confidence interval; CRE, carbapenem-resistant *Enterobacteriaceae*; cUTI, complicated urinary tract infection; DIC, disseminated intravascular coagulation; HABP, hospital-acquired bacterial pneumonia; IQR, interquartile range; IV, intervenous; NA, not applicable; OR, odds ratio; SBP, systolic blood pressure; SD, standard deviation; VABP, ventilator-associated bacterial pneumonia.

^a^Source of bacteremia was IV catheter in 47 of 140 (33.6%) patients, unknown in 23 of 140 (16.4%) patients, cUTI/AP in 12 of 140 (8.6%) patients, HABP in 5 of 140 (3.6%) patients, VABP in 5 of 140 (3.6%) patients, and “other” in 48 of 140 (34.3%) patients.

^b^Duration of hospitalization before index CRE infection.

^c^Immunocompromised condition included hematologic malignancy, prior bone marrow transplant, or received immunosuppressive therapy, such as cancer chemotherapy, antirejection medications for transplantation, or long-term (≥2 weeks) use of systemic steroids.

^d^Neutropenia was defined as <500 neutrophils/mm^3^.

^e^Although a patient may have had 2 or more different types of transplantations, the same patient was only counted once in the prior transplantations category.

^f^Chronic renal insufficiency was defined as moderate-to-severe renal disease.

^g^Empiric therapy without in vitro activity was defined as empiric antimicrobial therapy that did not contain at least 1 agent with in vitro activity against the index CRE pathogen according to microbiologic data entered.

^h^Severe sepsis was defined as infection associated with any of the following: hypotension (SBP ≤90 mmHg or a decrease in SBP of ≥40 mmHg from baseline [if known] unresponsive to fluid challenge), hypothermia (core temperature <35.6°C or <96.1°F), or DIC as evidenced by prothrombin time or partial thromboplastin time 2× the upper limit of normal or platelets less than 50% of the lower limit of normal [13].

^i^Septic shock (a subset of severe sepsis) was defined as infection associated with hypotension (SBP ≤90 mmHg or a decrease in SBP of ≥40 mmHg from baseline [if known] unresponsive to fluid challenge) [13].

^j^Prior duration of hospitalization greater than or equal to 14 days (OR, 1.89; 95% CI, 1.04–3.46), history of heart failure (OR, 2.41; 95% CI, 1.20–4.82), and presentation with severe sepsis (OR, 2.72; 95% CI, 1.50–4.96) were associated with increased likelihood of 28-day mortality on multivariate analysis. See Supplemental Table E.

### Comorbid Conditions

A substantial degree of comorbid conditions existed among the patient population, with a median Charlson Comorbidity Index of 3 (interquartile range, 2.0–5.0). The most frequent comorbidities included moderate-to-severe chronic renal insufficiency in 32.8% (84 of 256) of patients, with 19.9% (51 of 256) of patients requiring hemodialysis, diabetes mellitus in 28.1% (72 of 256), and solid tumor in 21.9% (56 of 256). Sixty-seven patients (26.2%) were immunocompromised, including 41 (16.0%) with prior transplantation (hematologic and solid organ). Illness severity at presentation was high among all patients; 84 of 256 (32.8%) patients presented with severe sepsis (sepsis accompanied by either shock, DIC, or hypothermia), and the average APACHE II score was 21.9 (standard deviation = 9.7). Among the 84 patients with severe sepsis, 75 (29.3% of the total study population) presented with septic shock. On analysis of comorbidities and risk factors, including prior CRE culture, diabetes, heart failure, chronic renal insufficiency, requirement for dialysis, solid tumor, hematologic malignancy, and hospitalization over 13 days, 112 of 256 (43.8%) patients had 3 or more covariates present. The most frequent covariates within the cohort were prior duration of hospitalization greater than 13 days (125 of 256, 48.6%), prior CRE culture (38.7%), and chronic renal insufficiency (32.8%) (Table 1; Supplemental Table A).

### Characteristics of Infecting Isolates

The most common index CRE pathogens were *Klebsiella* spp (224 of 256, 87.5%) and *Enterobacter* spp (15 of 256, 5.9%) (Tables 2 and 3). When identified, the mechanism of carbapenem resistance was production of the KPC enzyme in 174 of 180 (96.7%) isolates, metallo-β-lactamase production in 5 isolates (2.8%), and OXA production in 1 isolate (0.6%) (Tables 4 and 5). More than 80% of the index CRE isolates were nonsusceptible (intermediate or resistant) to penicillins, cephalosporins, aztreonam, fluoroquinolones, and trimethoprim-sulfamethoxazole. More than 80% (81.6%) of isolates were also nonsusceptible to tobramycin, 57.8% nonsusceptible to amikacin, 43.1% nonsusceptible to gentamicin, 37.0% nonsusceptible to tigecycline, and 26.4% nonsusceptible to colistin or polymyxin B (Supplemental Table B). Differences in antimicrobial susceptibility rates according to region are shown in Supplemental Table I.

**Table 2.** Microbiology of Index CRE Isolate by Type of Infection

Pathogen Name	Bacteremia (*n* = 140) *n* (%)	HABP (*n* = 21) *n* (%)	VABP (*n* = 20) *n* (%)	cUTI (*n* = 75) *n* (%)	All (*n* = 256) *n* (%)
*Klebsiella pneumoniae*	127 (90.7%)	18 (85.7%)	18 (90.0%)	59 (78.7%)	222 (86.7%)
*Enterobacter cloacae*	6 (4.3%)	1 (4.8%)	1 (5.0%)	4 (5.3%)	12 (4.7%)
*Escherichia coli*	3 (2.1%)	0	0	4 (5.3%)	7 (2.7%)
*Proteus mirabilis*	1 (0.7%)	0	0	4 (5.3%)	5 (2.0%)
*Serratia marcescens*	1 (0.7%)	1 (4.8%)	1 (5.0%)	1 (1.3%)	4 (1.6%)
*Enterobacter aerogenes*	1 (0.7%)	1 (4.8%)	0	1 (1.3%)	3 (1.2%)
*Klebsiella oxytoca*	1 (0.7%)	0	0	1 (1.3%)	2 (0.8%)
*Citrobacter freundii*	0	0	0	1 (1.3%)	1 (0.4%)

Abbreviations: CRE, carbapenem-resistant *Enterobacteriaceae*; cUTI, complicated urinary tract infection; HABP, hospital-acquired bacterial pneumonia; VABP, ventilator-associated bacterial pneumonia.

**Table 3.** Resistance Mechanisms of Infecting Isolate by Pathogen Type

Pathogen Name	KPC	MBL	OXA	Unknown^a^	All
*Klebsiella pneumoniae*	167^b^	5^b^	1	50	223
*Enterobacter cloacae*	2	0	0	10	12
*Escherichia coli*	1	0	0	6	7
*Proteus mirabilis*	0	0	0	5	5
*Serratia marcescens*	3	0	0	1	4
*Enterobacter aerogenes*	1	0	0	2	3
*Klebsiella oxytoca*	0	0	0	2	2
*Citrobacter freundii*	0	0	0	1	1
Total	174^b^	5^b^	1	77	257^b^

Abbreviations: CRE, carbapenem-resistant *Enterobacteriaceae*; KPC, *K pneumonia* carbapenemase; MBL, metallo-β-lactamase; OXA, OXA carbapenemases.

^a^Unknown, mechanism not determined (ie, no molecular testing performed).

^b^Patient 506NH-202-002 had 2 mechanisms of resistance (KPC and MBL) identified for the index CRE isolate (*K pneumoniae*).

**Table 4.** Empiric Antimicrobial Agents Stratified by Site of Infection

	Number (%) of Patients				
Empiric Antimicrobial Therapy	Infection Type				
	Bacteremia (*N* = 140)	HABP (*N* = 21)	VABP (*N* = 20)	cUTI (*N* = 75)	All (*N* = 256)
	*n* (%)	*n* (%)	*n* (%)	*n* (%)	*n* (%)
No treatment	8 (5.7%)	2 (9.5%)	0	18 (24.0%)	28 (10.9%)
No Gram-negative coverage	1 (0.7%)	0	1 (5.0%)	1 (1.3%)	3 (1.2%)
Gram-negative therapy	131 (93.6%)	19 (90.5%)	19 (95.0%)	56 (74.7%)	225 (87.9)
Monotherapy	72 (51.4%)	15 (71.4%)	14 (70.0%)	47 (62.7%)	148 (57.8)
All combination therapy	59 (42.1%)	4 (19.0%)	5 (25.0%)	9 (12.0%)	77 (30.1%)
Dual therapy	38 (27.1%)	1 (4.8%)	3 (15.0%)	7 (9.3%)	49 (19.1%)
3-drug combinations	19 (13.6%)	1 (4.8%)	2 (10.0%)	1 (1.3%)	23 (9.0%)
4 and more drug combinations	2 (1.4%)	2 (9.5%)	0	1 (1.3%)	5 (2.0%)
Number Active Agents					
No active agent	71 (50.7%)	15 (71.4%)	15 (75.0%)	41 (54.7%)	142 (55.5%)
One active agent	48 (34.3%)	3 (14.3%)	4 (20%)	15 (20.0%)	70 (27.3%)
** **Two active agents^a^	12 (8.6%)	1 (4.8%)	0	0	13 (5.1%)

Abbreviations: cUTI, complicated urinary tract infection; HABP, hospital-acquired bacterial pneumonia; VABP, ventilator-associated bacterial pneumonia.

^a^No subjects received empiric antimicrobial therapy with more than 2 agents with antimicrobial activity against the index CRE pathogen.

**Table 5.** Directed Antimicrobial Agents Stratified by Site of Infection

	Number (%) of Patients				
Directed Antimicrobial Therapy	Infection Type				
	Bacteremia (*N* = 140)	HABP (*N* = 21)	VABP (*N* = 20)	cUTI (*N* = 75)	All (*N* = 256)
	*n* (%)	*n* (%)	*n* (%)	*n* (%)	*n* (%)
No treatment	15 (10.7%)	3 (14.3%)	1 (5.0%)	21 (28.0%)	40 (15.6%)
No Gram-negative coverage	0	1 (4.8%)	1 (5.0%)	0	2 (0.8%)
Gram-negative therapy	125 (89.3%)	17 (81.0%)	18 (90.0%)	54 (72.0%)	214 (83.6%)
Monotherapy	33 (23.6%)	6 (28.6%)	3 (15.0%)	38 (50.7%)	80 (31.3%)
All combination therapy	92 (65.7%)	11 (52.4%)	15 (75.0%)	16 (21.3%)	134 (52.3%)
Dual therapy	40 (28.6%)	6 (28.6%)	5 (25.0%)	6 (8.0%)	57 (22.3%)
3-drug combinations	42 (30.0%)	4 (19.0%)	9 (45.05)	8 (10.7%)	63 (24.6%)
4 and more drug combinations	10 (7.1%)	1 (4.8%)	1 (5.0%)	2 (2.7%)	14 (5.5%)
Number Active Agents					
No active agent	18 (12.9%)	3 (14.3%)	4 (20.0%)	21 (28.0%)	46 (18.0%)
One active agent	70 (50.0%)	7 (33.3%)	8 (40.0%)	30 (40.0%)	115 (44.9%)
Two or more active agents	37 (26.4%)	7 (33.3%)	6 (30.0%)	3 (4.0%)	53 (20.7%)
Two active agents	33 (23.6%)	7 (33.3%)	5 (25.0%)	3 (4.0%)	48 (18.8%)
Three active agents	3 (2.1%)	0	1 (5.0%)	0	4 (1.6%)
Four or more active agents	1 (0.7%)	0	0	0	1 (0.7%)

Abbreviations: cUTI, complicated urinary tract infection; HABP, hospital-acquired bacterial pneumonia; VABP, ventilator-associated bacterial pneumonia.

### Antimicrobial Treatment

Among the 256 patients included, a majority (148 of 256, 57.8%) received empiric therapy with antibiotic active against Gram-negative pathogens. Of the 4 infection types, bacteremic patients were most likely to receive empiric therapy with 2 or more agents with Gram-negative activity (59 of 256, 42.1%) (Table 4). Despite this, only 83 subjects (32.4%) received empiric coverage with agents that had in vitro activity against the index CRE isolate. The most common classes of empiric antimicrobials across all cases (both as mono- and combination therapy) were β-lactam/β-lactamase inhibitor combinations (eg, piperacillin-tazobactam, ampicillin-sulbactam) (91 of 228, 39.9%); carbapenems (89 of 228, 39.0%); and aminoglycosides (eg, gentamicin, amikacin, or tobramycin) (68 of 228, 29.8%) (Supplemental Table C).

One hundred sixty-eight of the 256 total patients (65.6%) ultimately received directed antimicrobial therapy with an agent that had in vitro activity against their CRE isolate. There were 69 different regimens used comprising of single or combination therapy with up to 4 agents. A majority of patients (134 of 256, 52.3%) received directed therapy with more than 1 Gram-negative agent: 57 of 134 (42.5%) patients received dual therapy and 77 of 134 (57.5%) patients received 3 or more antimicrobials with Gram-negative activity. Among the 168 patients whose directed therapy included an antibacterial with in vitro activity against their CRE pathogen, 115 (68.5%) received only 1 active agent, 48 of 168 (28.6%) received 2 active agents, and 5 of 168 (3.0%) received 3 or more active agents (Table 5).

The most common directed antimicrobials by therapeutic class across all cases (both as mono- and combination therapy) were polymyxins (101 of 256, 39.5%), carbapenems (97 of 256, 37.9%), tigecycline (95 of 256, 37.1%), and aminoglycosides (eg, gentamicin, amikacin, and tobramycin) (90 of 256, 35.2%). Despite a concerted effort, we were unable to identify a single regimen consistent with best available therapy due to the large number of different regimens used in the treatment of CRE infections (Supplemental Table D).

### Outcomes

Approximately half (57.0%) of patients achieved clinical cure, and 134 of 256 (52.3%) achieved microbiologic eradication at the completion of antibiotic treatment (Table 6). Overall, 72 of the 256 patients (28.1%) died within 28 days of the index CRE infection. Across all infection types, risk factors associated with 28-day mortality included a duration of hospitalization over 13 days (OR, 1.89; 95% CI, 1.04–3.46), presence of underlying heart failure (OR, 2.41; 95% CI, 1.20–4.83), and presentation with severe sepsis (including septic shock) (OR, 2.72; 95% CI, 1.50–4.96) (Supplemental Table E). There were no statistically significant differences in either clinical cure or mortality rates according to the type of empiric antimicrobial therapy used—combination therapy or monotherapy. This was true regardless of whether combination therapy was defined by more than 1 Gram-negative agent or more than 1 agent with in vitro activity against the index CRE pathogen. Although directed antimicrobial therapy with more than 1 agent with in vitro activity against the index CRE pathogen was associated with a higher percentage of clinical cures (69.8% vs 57.4%; OR, 1.58; 95% CI, 0.78–3.17) and lower 28-day mortality (20.8% vs 27.0%; OR, 0.62; 95% CI, 0.28–1.37), neither of these reached statistical significance (Supplemental Table F).

**Table 6.** Outcomes of Infections Due to Carbapenem-Resistant *Enterobacteriaceae* by Type of Infection and Overall

Outcome	Number (%) of Patients				
	Infection Type				
	Bacteremia (*N* = 140)	HABP (*N* = 21)	VABP (*N* = 20)	cUTI/AP (*N* = 75)	All (*N* = 256)
Duration of hospitalization for index CRE infection^b^ (mean ± SD)	17.9 (17.5)	11.7 (7.2)	12.4 (6.4)	8.2 (12.7)	14.1 (15.4)
Duration of ICU stay^b^ (mean ± SD)	9.5 (15.8)	7.7 (16.1)	14.1 (12.0)	3.6 (11.6)	8.0 (14.7)
Number (%) With Clinical Cure (Study Investigator-Ascertained)					
** **Yes	74 (52.9%)	9 (42.9%)	9 (45%)	54 (72.0%)	146 (57.0%)
** **No	62 (44.3%)	11 (52.4%)	10 (50.0%)	19 (25.3%)	102 (39.8)
** **Unknown	4 (2.9%)	1 (4.8%)	1 (5.0%)	2 (2.7%)	8 (3.1%)
CRE Organism Eradicated From Site of Infection, n (%)					
** **Yes	78 (55.7%)	8 (38.1%)	10 (50.0%)	38 (50.7%)	134 (52.3%)
** **No	51 (36.4%)	10 (47.6%)	9 (45.0%)	29 (38.7%)	99 (38.7%)
** **Unknown	11 (7.9%)	3 (14.3%)	1 (5.0%)	8 (10.7%)	23 (9.0)
28-Day Mortality, n (%)					
** **Yes	45 (32.1%)	7 (33.3%)	7 (35.0%)	13 (17.3%)^a^	72 (28.1%)
** **No	82 (58.6%)	12 (57.1%)	12 (60%)	51 (68.0%)	157 (61.3%)
** **Unknown	13 (9.3%)	2 (9.5%)	1 (5.0%)	11 (14.7%)	27 (10.5%)

Abbreviations: AP, acute pyelonephritis; CRE, carbapenem-resistant *Enterobacteriaceae*; cUTI, complicated urinary tract infection; HABP, hospital-acquired bacterial pneumonia; ICU, intensive care unit; PI, ; SD, standard deviation; VABP, ventilator-associated bacterial pneumonia.

^a^Mortality rate among patients with cUTI/AP with concurrent bacteremia was 22.2%.

^b^Duration of hospitalization for index CRE infection refers to the duration of hospitalization required for treatment of the index CRE infection after diagnosis. Duration of ICU stay refers to the duration of ICU stay required for treatment of the index CRE infection.

Table 7 shows outcomes of CRE infections within patient subgroups that are often excluded in phase 3 clinical trials of new antimicrobial agents, including patients with moderate-to-severe chronic renal insufficiency [22–26, 33], those requiring dialysis [22–28, 33], those with immunocompromised status [22–26, 33], and patients who presented with severe sepsis [22–26, 33]. Among patients with chronic renal insufficiency, the 28-day mortality rate was 33.3%, which increased to 41.2% in those requiring dialysis. Among patients with an immunocompromised condition, the 28-day mortality rate was 31.3%. Among patients who presented with evidence of severe sepsis (32.8% of all cases) and septic shock (29.3% of all cases), the 28-day mortality rates were 44.0% and 46.7%, respectively.

**Table 7.** Outcomes of Infections Due to Carbapenem-Resistant *Enterobacteriaceae* Within Specific Subgroups

Subgroup	Number, (%)^a^ of Patients						
	28-Day Mortality n (%)			Clinical Cure^b^ n (%)			Total in Subgroup
	Yes	No	Unknown	Yes	No	Unknown	n/N (%)^c^
Chronic renal insufficiency^d^	28 (33.3%)	49 (58.3%)	7 (8.3%)	41 (48.8%)	41 (48.8%)	2 (2.4%)	84 (32.8%)
Requirement for dialysis	21 (41.2%)	26 (51.0%)	4 (7.8%)	18 (35.3%)	30 (58.8%)	3 (5.9%)	51 (19.8%)
Immunocompromised condition^e^	21 (31.3%)	41 (61.2%)	5 (7.5%)	34 (50.7)	33 (49.3%)	0 (0.0%)	67 (26.2%)
Presentation with severe sepsis^f^	37 (44.0%)	40 (47.6%)	7 (8.3%)	37 (44.0%)	45 (53.6%)	2 (2.4%)	84 (32.8%)
Presentation with septic shock^g^	35 (46.7%)	35 (46.7%)	5 (6.7%)	31 (41.3%)	42 (56.0%)	2 (2.7%)	75 (29.3%)

Abbreviations: DIC, disseminated intravascular coagulation; SBP, systolic blood pressure.

^a^n represents the number of patients in each subgroup who met the respective endpoint. The percentage (%) of subjects in that subgroup who met that endpoint is denoted below the value for n (eg, 28 subjects with chronic renal insufficiency died by day 28. This is 33.3% of the total number of subjects with chronic renal insufficiency).

^b^Clinical cure as ascertained by study investigator.

^c^n represents the number and percentage (%) of patients with each condition within the total study population (*N***=** 256).

^d^Chronic renal insufficiency was defined as moderate-to-severe renal disease.

^e^Immunocompromised condition included hematologic malignancy, prior bone marrow transplant, or received immunosuppressive therapy, such as cancer chemotherapy, antirejection medications for transplantation, or long-term (≥2 weeks) use of systemic steroids.

^f^Severe sepsis was defined as infection associated with any of the following: hypotension (SBP ≤90 mmHg or a decrease in SBP of ≥40 mmHg from baseline [if known] unresponsive to fluid challenge), hypothermia (core temperature <35.6°C or <96.1°F), or DIC as evidenced by prothrombin time or partial thromboplastin time 2× the upper limit of normal or platelets less than 50% of the lower limit of normal [13].

^g^Septic shock (a subset of severe sepsis) was defined as infection associated with hypotension (SBP ≤90 mmHg or a decrease in SBP of ≥40 mmHg from baseline [if known] unresponsive to fluid challenge) [13].

## DISCUSSION

Carbapenem-resistant *Enterobacteriaceae* infections are associated with a high 28-day overall mortality (28.1%), low rate of clinical cure (57.0%), and low rate of microbiologic eradication (52.3%) [15, 29–32]. In our analysis, mortality was lowest in patients with cUTI/AP, yet still striking, at 17.3%, compared with much lower mortality rates (ranging from 0% to 2%) in recent registration clinical trials of patients with cUTI/AP [22, 24, 33]. Mortality rates were higher among immunocompromised patients, those with moderate-to-severe renal insufficiency (especially those on dialysis), and patients who presented with severe sepsis. Although most patients received combination antimicrobial therapy, including agents with activity against the isolate, this did not have a statistically significant benefit with regards to survival or clinical cure. Thus, outcomes of CRE infections even in patients treated with individualized best available therapy of single or combination agents are poor, which is consistent with previous studies [12, 15, 29–31]. The findings of this analysis have multiple implications for the design of clinical trials of new antimicrobials targeted for treating resistant organisms such as CRE. Most notably, they highlight the high degree of comorbidities and index severity with which patients with CRE infections present. Almost one half of patients (48.6%) had been hospitalized for over 13 days at the time of CRE infection, almost one third of patients (32.8%) had chronic renal insufficiency, and more than one quarter of the patients (26.2%) were immunocompromised. Illness severity at presentation is highlighted by the high rate of presentation with severe sepsis (32.8%), which ranged from 20% (cUTI/AP) to 55% (VABP), and septic shock, which ranged from 18.7% (cUTI/AP) to 40% (VABP). The substantial comorbidity in this patient population presents a major challenge in designing clinical trials of such new agents, particularly with regards to the selection of inclusion and exclusion criteria, because most recent registration clinical trials of new antimicrobial agents have excluded patients with underlying immune compromise [23–26, 33], moderate-to-severe renal insufficiency [23–26, 33], need for dialysis [23–28, 33], or presentation with severe sepsis [23–26, 33]. Given the high prevalence of these conditions in the population with CRE infections, exclusion of these subgroups in a CRE-targeting clinical trial would greatly limit recruitment. Indeed, in reviewing the inclusion and exclusion criteria originally proposed for a phase 3 clinical study of meropenem-vaborbactam in patients with CRE infections (Clinical Trial NCT02168946) [7] against the results of this study, it was noted that only 22% of subjects in this retrospective analysis would have been eligible for enrollment. In light of these data, the phase 3 trial was amended to allow inclusion of subjects with these conditions (immunocompromised, renal insufficiency including need for dialysis, and severe sepsis), resulting in a significant increase in enrollment. The high rate of mortality seen in this study implies that clinical trials in patients with CRE infections are likely to have similarly high mortality rates, in contrast to the low mortality rates seen in recent indication-specific clinical trials [23, 24, 33]. Finally, the high rate of resistance to antimicrobial agents commonly used against CRE pathogens (eg, polymyxins) and lack of clear consensus in antimicrobial regimens for CRE infections underscores the lack of options available for treating these infections and the pressing need for new antimicrobial agents. Indeed, one reason for the lack of a statistically significant benefit to combination therapy with more than 1 in vitro active agent may have been the small percentage of subjects (53 of 256, 20.7%) who received directed therapy with 2 or more active agents. In contrast, in previous studies where a benefit to combination therapy was found, more than 50% of patients received 2 or more active drugs [29, 30, 34–36]. This change may reflect increasing resistance to agents commonly used as combination therapy against CRE pathogens [37, 38].

This study has several limitations. Similar to other retrospective studies of CRE infections [29–31], stringent definitions of infection could not be applied. Although use of regulatory guidance-based definitions would have minimized this, it would have impeded the objective of informing clinical trial inclusion criteria. In addition, the use of the date of CRE culture availability to mark the beginning of directed therapy may have led to misclassification of empiric therapy as directed. Although other definitions were considered, these were rejected, because they were more likely to reflect antibiotic changes due to clinical failures. Finally, this study did not examine antimicrobial use within the 3 months before treatment of the index infection, which other studies have found to be a risk factor for CRE infection [15].

## CONCLUSIONS

Regulatory guidance-directed clinical trials allow a consistent, well characterized patient population for pursuing registration studies of new agents [3]. The patient population of these trials is identified according to conventional inclusion and exclusion criteria for the specified infection type or indication. This approach enables comparisons of different agents across different trials and over time (assuming a stationary effect of the control agent in noninferiority studies). For clinical trials of agents directed at drug-resistant pathogens such as CRE, a trial based on standard enrollment criteria in patients at low risk of infection due to these pathogens would not inform on the efficacy or safety of the novel agent in the target patient population at risk. In light of these considerations, we have used the results of this study to inform the design of a prospective, randomized, multinational trial comparing meropenem-vaborbactam to best available therapy in patients with CRE infections [7]. Such studies are important to provide supportive information on the efficacy, pharmacokinetics/dosing, and safety of new agents in the target population being treated for resistant pathogens where options are limited [39, 40]. Moreover, such trials empower future stewardship efforts maintained at preserving efficacy of new agents targeting resistant pathogens [39]. Thus, observational trials in these target populations are a useful means to inform on the design of clinical trials while also providing historical control for those investigations.

## Supplementary Data

Supplementary materials are available at *Open Forum Infectious Diseases* online. Consisting of data provided by the authors to benefit the reader, the posted materials are not copyedited and are the sole responsibility of the authors, so questions or comments should be addressed to the corresponding author.

## Supplementary Material

ofx063_suppl_Supplemental_Tables_AtoI_March14_FINAL
