# Supplementary material for: Carbapenem-Resistant Enterobacteriaceae Infections: Results From a Retrospective Series and Implications for the Design of Prospective Clinical Trials
Source: Open Forum Infect Dis. 2017 Jun 1;4(2):ofx063. doi: 10.1093/ofid/ofx063 (PMC5451664; doi:10.1093/ofid/ofx063)
Supplement: ofx063_suppl_Supplemental_Tables_AtoI_March14_FINAL [file ofx063_suppl_supplemental_tables_atoi_march14_final.docx]

| **Supplemental Table A.** Frequency of Covariates and Covariate Patterns Across the Cohort of Patients with CRE Infections | |
| --- | --- |
| **Number of Covariates*** | **All Cases (N=256)** |
| 0 | 27 (10.5) |
| 1 | 54 (21.0%) |
| 2 | 63 (24.6%) |
| 3 | 68 (26.6%) |
| 4 or More | 44 (17.2%) |
| Frequency of Common Covariates |  |
| Prior duration of hospitalization >13 days | 125 (48.6%) |
| Prior Culture Positive for CRE and prior hospitalization >13 days | 62 (24.2%) |
| Chronic Renal Insufficiency, Prior Culture Positive for CRE and  Prior hospitalization >13 days | 20 (7.8%) |

* Covariates examined included: prior positive cultures for CRE, presence of diabetes mellitus, heart failure, chronic renal insufficiency, requirement for dialysis, solid tumor, hematologic malignancy, and duration of hospitalization greater 13 days.

| Supplemental Table B. Summary of Antimicrobial Non-Susceptibility Results by Infection Type (with ≥10 Isolates) – All CRE Cases | | | | | | | | | | | | | | | |
| --- | --- | --- | --- | --- | --- | --- | --- | --- | --- | --- | --- | --- | --- | --- | --- |
|  | Infection Type | | | | | | | | | | | | | | |
|  | cUTI/  AP | | | HABP | | | VABP | | | Bacteremia | | | All Cases^1^ | | |
| Antibiotic Test | N^2^ | %  Sus | % Non- Sus^3^ | N^2^ | % Sus | % Non- Sus^3^ | N^2^ | % Sus | % Non- Sus^3^ | N^2^ | % Sus | % Non- Sus^3^ | N^2^ | % Sus | % Non- Sus^3^ |
| **Ampicillin/ sulbactam** | 2/  0 | - | 100.0/  - | 2 | - | 100.0 | 3 | - | 100.0 | 22 | - | 100.0 | 29 | - | 100.0 |
| **Cefazolin** | 3/  0 | - | 100.0/  - | 0 | - | - | 2 | - | 100.0 | 13 | - | 100.0 | 18 | - | 100.0 |
| **Cephalothin** | 1/  0 | - | 100.0/  - | 2 | - | 100.0 | 2 | - | 100.0 | 9 | - | 100.0 | 14 | - | 100.0 |
| **Ertapenem** | 37/  3 | 8.1/  - | 91.9/ 100.0 | 14 | - | 100.0 | 16 | - | 100.0 | 85 | 1.2 | 98.8 | 156 | 2.6 | 97.4 |
| **Ceftriaxone** | 6/  0 | - | 100.0/  - | 2 | 50.0 | 50.0 | 4 | - | 100.0 | 24 | - | 100.0 | 36 | 2.8 | 97.2 |
| **Meropenem** | 66/  7 | 9.0/  - | 91.0/  100.0 | 19 | - | 100.0 | 18 | - | 100.0 | 128 | 1.6 | 98.4 | 239 | 3.3 | 96.7 |
| **Aztreonam** | 44/  4 | 2.3/  - | 97.7/ 100.0 | 11 | 18.2 | 81.8 | 13 | - | 100.0 | 58 | 3.4 | 96.6 | 130 | 3.8 | 96.2 |
| **Imipenem** | 35/  4 | 8.6/  - | 91.4/ 100.0 | 10 | 10.0 | 90.0 | 14 | - | 100.0 | 81 | 2.5 | 97.5 | 145 | 4.1 | 95.9 |
| **Ceftazidime** | 33/  0 | 3.0/  - | 97.0/  - | 14 | 14.3 | 85.7 | 9 | - | 100.0 | 92 | 3.3 | 96.7 | 148 | 4.1 | 95.9 |
| **Piperacillin-tazobactam** | 52/  4 | 9.7/  - | 90.3/ 100.0 | 17 | 11.8 | 88.2 | 14 | - | 100.0 | 123 | 3.3 | 96.7 | 211 | 5.2 | 94.8 |
| **Cefuroxime** | 1/  0 | - | 100.0/  - | 2 | 50.0 | 50.0 | 2 | - | 100.0 | 11 | - | 100.0 | 16 | 6.3 | 93.8 |
| **Cefotaxime** | 5/  0 | - | 100.0/  - | 0 | - | - | 0 | - | - | 6 | 16.7 | 83.3 | 11 | 9.1 | 90.9 |
| **Ciprofloxacin** | 50/  5 | 14.0/ 20.0 | 86.0/ 80.0 | 17 | 23.5 | 76.5 | 13 | 7.7 | 92.3 | 118 | 9.3 | 90.7 | 203 | 11.8 | 88.2 |
| **Tobramycin** | 56/  5 | 25.0/ 40.0 | 75.0/ 60.0 | 9 | 22.2 | 77.8 | 11 | 9.1 | 90.9 | 60 | 11.7 | 88.3 | 141 | 18.4 | 81.6 |
| **Levofloxacin** | 23/  2 | 30.4/  - | 69.6/ 100.0 | 5 | 20.0 | 80.0 | 7 | 14.3 | 85.7 | 38 | 13.2 | 86.8 | 75 | 18.7 | 81.3 |
| **Sulfamethoxazole/ trimethoprim** | 5/  0 | 60.0/  - | 40.0/  - | 2 | 50.0 | 50.0 | 4 | 25.0 | 75.0 | 24 | 20.8 | 79.2 | 35 | 28.6 | 71.4 |
| **Cefepime** | 6/  0 | 50.0/  - | 50.0/  - | 5 | 80.0 | 20.0 | 4 | 50.0 | 50.0 | 29 | 27.6 | 72.4 | 44 | 38.6 | 61.4 |
| **Fosfomycin** | 9/  0 | 44.4/  - | 55.6/  - | 0 | - | - | 0 | - | - | 10 | 40.0 | 60.0 | 19 | 42.1 | 57.9 |
| **Amikacin** | 58/  3 | 74.1/ 100.0 | 25.9/  - | 19 | 47.4 | 52.6 | 17 | 17.6 | 82.4 | 126 | 28.6 | 71.4 | 223 | 42.2 | 57.8 |
| **Gentamicin** | 67/  7 | 53.7/ 100.0 | 46.3/  - | 21 | 71.4 | 28.6 | 20 | 65.0 | 35.0 | 139 | 53.2 | 46.8 | 255 | 56.9 | 43.1 |
| **Tigecycline** | 24/  1 | 62.5/ 100.0 | 37.5/  - | 15 | 86.7 | 13.3 | 11 | 54.5 | 45.5 | 110 | 60.9 | 39.1 | 162 | 63.0 | 37.0 |
| **Temocillin** | 1/  0 | 100.0/  - | - | 0 | - | - | 0 | - | - | 10 | 60.0 | 40.0 | 11 | 63.6 | 36.4 |
| **Colistin** | 25/  4 | 76.0/ 100.0 | 24.0/  - | 11 | 81.8 | 18.2 | 12 | 75.0 | 25.0 | 104 | 71.2 | 28.8 | 157 | 73.2 | 26.8 |
| **Polymyxin B** | 7/  0 | 100.0/  - | - | 4 | 100.0 | - | 1 | 100.0 | - | 9 | 77.8 | 22.2 | 21 | 90.5 | 9.5 |
| Note: The dash symbol (-) represents isolates that were not tested by the corresponding antibiotic.  ^1^Antibiotic tests are presented in descending order by percentage of non-susceptibility results in the All Cases column.  ^2^N was defined as the number of isolates.  ^3^% Non-susceptible equals % Resistant + % Intermediate (where either or both criteria are available), or 100% - % Susceptible (where neither Resistant nor Intermediate criteria are available).  AP = acute pyelonephritis; CRE = carbapenem-resistant Enterobacteriaceae; cUTI = complicated urinary tract infection; HABP = hospital-acquired bacterial pneumonia; Non-sus = non-susceptible; Sus = susceptible; VABP = ventilator-associated bacterial pneumonia  Sources: Post-text Tables 2.6.4.1 and 2.6.4.2 | | | | | | | | | | | | | | | |

| **Supplemental Table C**   Empiric Antimicrobial Regimens Stratified by Site of Infection and Region of Origin (All Cases) | | | | | | | | |
| --- | --- | --- | --- | --- | --- | --- | --- | --- |
|  | **Bacteremia** | | **HABP** | | **VABP** | | **cUTI/AP** | |
| **Empiric Antimicrobial Therapy** | **US (N=50) n (%)** | **EU (N=90) n (%)** | **US (N=11) n (%)** | **EU (N=10) n (%)** | **US (N=10) n (%)** | **EU (N=10) n (%)** | **US (N=61) n (%)** | **EU (N=14) n (%)** |
| **Monotherapy** | **9 (18·0)** | **33 (36·7)** | **5 (45·5)** | **5 (50·0)** | **2 (20·0)** | **7 (70·0)** | **22 (36·1)** | **7 (50.0)** |
| Beta-lactamase | 1 (2·0) | 11 (12·2) | 3 (27·3) | 1 (10·0) | 0 | 6 (60·0) | 10 (16·4) | 0 |
| Carbapenem | 3 (6·0) | 9 (10·0) | 1 (9·1) | 3 (30·0) | 1 (10·0) | 0 | 1 (1·6) | 2 (14·3) |
| Fluroquinolone | 1 (2·0) | 3 (3·3) | 0 | 0 | 0 | 0 | 3 (4·9) | 1 (7.1) |
| Aminoglycoside | 1 (2·0) | 2 (2·2) | 0 | 0 | 0 | 0 | 1 (1·6) | 2 (14.3)) |
| Cephalosporin (3rd) | 1 (2·0) | 2 (2·2) | 0 | 0 | 0 | 0 | 3 (4·9) | 1 (7.1) |
| Cephalosporin (4th) | 2 (4·0) | 0 | 0 | 0 | 1 (10·0) | 0 | 2 (3·3) | 0 |
| Penicillin + beta-lactamase inhibitor | 0 | 2 (2·2) | 1 (9·1) | 1 (10·0) | 0 | 0 | 0 | 0 |
| Polymyxin | 0 | 0 | 0 | 0 | 0 | 1 (10·0) | 1 (1·6) | 1 (7.1) |
| Tigecycline | 0 | 3 (3·3) | 0 | 0 | 0 | 0 | 0 | 0 |
| Monobactam | 0 | 1 (1·1) | 0 | 0 | 0 | 0 | 1 (1·6) | 0 |
| **Dual Therapy** | **18 (36·0)** | **23 (25·6)** | **2 (18·2)** | **3 (30·0)** | **5 (50·0)** | **1 (10·0)** | **14 (23·0)** | **3 (21.4)** |
| Aminoglycoside, Carbapenem | 2 (4·0) | 7 (7·8) | 0 | 0 | 0 | 0 | 1 (1·6) | 0 |
| Beta-lactamase, Fluroquinolone | 3 (6·0) | 2 (2·2) | 0 | 1 (10·0) | 0 | 1 (10·0) | 1 (1·6) | 0 |
| Aminoglycoside, Beta-lactamase | 1 (2·0) | 2 (2·2) | 0 | 0 | 1 (10·0) | 0 | 2 (3·3) | 0 |
| 1st Generation Cephalosporin, Beta-lactamase | 1 (2·0) | 0 | 1 (9·1) | 0 | 1 (10·0) | 0 | 2 (3·3) | 0 |
| Aminoglycoside, Cephalosporin (4th) | 3 (6·0) | 1 (1·1) | 0 | 0 | 0 | 0 | 0 | 0 |
| Carbapenem, Polymyxin | 0 | 2 (2·2) | 0 | 0 | 2 (20·0) | 0 | 0 | 0 |
| Beta-lactamase, Carbapenem | 1 (2·0) | 0 | 1 (9·1) | 0 | 0 | 0 | 0 | 1 (7.1)) |
| Carbapenem, Tigecycline | 0 | 2 (2·2) | 0 | 1 (10·0) | 0 | 0 | 0 | 0 |
| Aminoglycoside, Cephalosporin (3rd) | 1 (2·0) | 0 | 0 | 0 | 0 | 0 | 1 (1·6) | 0 |
| Beta-lactamase, Cephalosporin (3rd) | 1 (2·0) | 0 | 0 | 0 | 0 | 0 | 1 (1·6) | 0 |
| Beta-lactamase, Polymyxin | 0 | 2 (2·2) | 0 | 0 | 0 | 0 | 0 | 0 |
| Carbapenem, Macrolide | 1 (2·0) | 0 | 0 | 0 | 0 | 0 | 1 (1·6) | 0 |
| Carbapenem, TMP/SMX | 0 | 2 (2·2) | 0 | 0 | 0 | 0 | 0 | 0 |
| Cephalosporin (3rd), TMP/SMX | 0 | 0 | 0 | 1 (10·0) | 0 | 0 | 1 (1·6) | 0 |
| Aminoglycoside, Fluroquinolone | 0 | 0 | 0 | 0 | 0 | 0 | 1 (1·6) | 0 |
| Aminoglycoside, Monobactam | 0 | 0 | 0 | 0 | 0 | 0 | 1 (1·6) | 0 |
| Aminoglycoside, Penicillin + beta-lactamase inhibitor | 0 | 1 (1·1) | 0 | 0 | 0 | 0 | 0 | 0 |
| Aminoglycoside, Polymyxin | 0 | 1 ( 1·1) | 0 | 0 | 0 | 0 | 0 | 0 |
| Aminoglycoside, Tigecycline | 0 | 1 ( 1·1) | 0 | 0 | 0 | 0 | 0 | 0 |
| Beta-lactamase, Penicillin + beta-lactamase inhibitor | 0 | 0 | 0 | 0 | 0 | 0 | 0 | 1 (7.1) |
| Beta-lactamase, Tigecycline | 1 (2·0) | 0 | 0 | 0 | 0 | 0 | 0 | 0 |
| Carbapenem, Fluroquinolone | 0 | 0 | 0 | 0 | 0 | 0 | 1 (1·6) | 0 |
| Cephalosporin (3rd), Cephalosporin (4th) | 0 | 0 | 0 | 0 | 0 | 0 | 1 (1·6) | 0 |
| Cephalosporin (3rd), Macrolide | 0 | 0 | 0 | 0 | 0 | 0 | 0 | 1 (7.1) |
| Fluroquinolone, Monobactam | 1 (2·0) | 0 | 0 | 0 | 0 | 0 | 0 | 0 |
| Fluroquinolone, Tigecycline | 1 (2·0) | 0 | 0 | 0 | 0 | 0 | 0 | 0 |
| Monobactam, TMP/SMX | 0 | 0 | 0 | 0 | 1 (10·0) | 0 | 0 | 0 |
| Polymyxin, Tigecycline | 1 (2·0) | 0 | 0 | 0 | 0 | 0 | 0 | 0 |
| **3-Drug Combinations** | **15 (30·0)** | **17 (18·9)** | **1 (9·1)** | **0** | **2 (20·0)** | **1 (10·0)** | **6 (9·8)** | 1 (7.1) |
| Aminoglycoside, Carbapenem, Tigecycline | 0 | 7 (7·8) | 0 | 0 | 0 | 0 | 1 (1·6) | 1 (7.1) |
| Carbapenem, Polymyxin, Tigecycline | 1 (2·0) | 4 (4·4) | 0 | 0 | 0 | 1 (10·0) | 1 (1·6) | 0 |
| Aminoglycoside, Beta-lactamase, Carbapenem | 2 (4·0) | 0 | 0 | 0 | 0 | 0 | 0 | 0 |
| Aminoglycoside, Carbapenem, Polymyxin | 0 | 2 (2·2) | 0 | 0 | 0 | 0 | 0 | 0 |
| Beta-lactamase, Carbapenem, Tigecycline | 2 (4·0) | 0 | 0 | 0 | 0 | 0 | 0 | 0 |
| 1st Generation Cephalosporin, Aminoglycoside, Beta-lactamase | 0 | 0 | 1 (9·1) | 0 | 0 | 0 | 0 | 0 |
| 1st Generation Cephalosporin, Aminoglycoside, Tigecycline | 1 (2·0) | 0 | 0 | 0 | 0 | 0 | 0 | 0 |
| Aminoglycoside, Beta-lactamase, Phosphonic | 0 | 1 (1·1) | 0 | 0 | 0 | 0 | 0 | 0 |
| Aminoglycoside, Beta-lactamase, Polymyxin | 1 (2·0) | 0 | 0 | 0 | 0 | 0 | 0 | 0 |
| Aminoglycoside, Beta-lactamase, Tigecycline | 0 | 1 (1·1) | 0 | 0 | 0 | 0 | 0 | 0 |
| Aminoglycoside, Carbapenem, Cephalosporin (3rd) | 1 (2·0) | 0 | 0 | 0 | 0 | 0 | 0 | 0 |
| Aminoglycoside, Carbapenem, Macrolide | 0 | 1 (1·1) | 0 | 0 | 0 | 0 | 0 | 0 |
| Aminoglycoside, Cephalosporin (3rd), Polymyxin | 1 (2·0) | 0 | 0 | 0 | 0 | 0 | 0 | 0 |
| Aminoglycoside, Cephalosporin (4th), Tigecycline | 0 | 0 | 0 | 0 | 1 (10·0) | 0 | 0 | 0 |
| Beta-lactamase, Carbapenem, Cephalosporin (4th) | 0 | 0 | 0 | 0 | 1 (10·0) | 0 | 0 | 0 |
| Beta-lactamase, Carbapenem, Penicillin + beta-lactamase inhibitor | 0 | 0 | 0 | 0 | 0 | 0 | 1 (1·6) | 0 |
| Beta-lactamase, Fluroquinolone, Monobactam | 1 (2·0) | 0 | 0 | 0 | 0 | 0 | 0 | 0 |
| Beta-lactamase, Fluroquinolone, Penicillin + beta-lactamase inhibitor | 1 (2·0) | 0 | 0 | 0 | 0 | 0 | 0 | 0 |
| Beta-lactamase, Penicillin + beta-lactamase inhibitor, TMP/SMX | 1 (2·0) | 0 | 0 | 0 | 0 | 0 | 0 | 0 |
| Carbapenem, Cephalosporin (3rd), Fluroquinolone | 1 (2·0) | 0 | 0 | 0 | 0 | 0 | 0 | 0 |
| Carbapenem, Cephalosporin (3rd), Polymyxin | 0 | 0 | 0 | 0 | 0 | 0 | 1 (1·6) | 0 |
| Carbapenem, Fluroquinolone, Polymyxin | 1 (2·0) | 0 | 0 | 0 | 0 | 0 | 0 | 0 |
| Cephalosporin (3rd), Fluroquinolone, Monobactam | 0 | 0 | 0 | 0 | 0 | 0 | 1 (1·6) | 0 |
| Cephalosporin (3rd), Fluroquinolone, Nitrofurantoins | 0 | 0 | 0 | 0 | 0 | 0 | 1 (1·6) | 0 |
| Fluroquinolone, Macrolide, Phosphonic | 0 | 1 (1·1) | 0 | 0 | 0 | 0 | 0 | 0 |
| Polymyxin, Rifabutin, Tigecycline | 1 (2·0) | 0 | 0 | 0 | 0 | 0 | 0 | 0 |
| **4 and more Drug Combinations** | **8 (16·0)** | **8 (8·9)** | **1 (9·1)** | **2 (20·0)** | **1 (10·0)** | **0** | **3 (4·9)** | **0** |
| Aminoglycoside, Beta-lactamase, Cephalosporin (4th), Tigecycline | 2 (4·0) | 0 | 0 | 0 | 0 | 0 | 0 | 0 |
| Aminoglycoside, Carbapenem, Polymyxin, Rifabutin, Tigecycline | 0 | 2 (2·2) | 0 | 0 | 0 | 0 | 0 | 0 |
| 1st Generation Cephalosporin, Beta-lactamase, Carbapenem, Cephalosporin (4th) | 1 (2·0) | 0 | 0 | 0 | 0 | 0 | 0 | 0 |
| 1st Generation Cephalosporin, Beta-lactamase, Cephalosporin (3rd), Macrolide, Monobactam, Tigecycline | 0 | 0 | 0 | 0 | 1 (10·0) | 0 | 0 | 0 |
| 1st Generation Cephalosporin, Carbapenem, Fluroquinolone, Penicillin + beta-lactamase inhibitor | 1 (2·0) | 0 | 0 | 0 | 0 | 0 | 0 | 0 |
| 2nd Generation Cephalosporin, Beta-lactamase, Cephalosporin (3rd), Fluroquinolone | 1 (2·0) | 0 | 0 | 0 | 0 | 0 | 0 | 0 |
| Aminoglycoside, Beta-lactamase, Carbapenem, Cephalosporin (3rd) | 0 | 0 | 0 | 0 | 0 | 0 | 1 (1·6) | 0 |
| Aminoglycoside, Beta-lactamase, Carbapenem, Macrolide, Penicillin + beta-lactamase inhibitor, TMP/SMX, Tigecycline | 0 | 0 | 1 (9·1) | 0 | 0 | 0 | 0 | 0 |
| Aminoglycoside, Beta-lactamase, Cephalosporin (3rd), Cephalosporin (4th), TMP/SMX | 0 | 1 (1·1) | 0 | 0 | 0 | 0 | 0 | 0 |
| Aminoglycoside, Carbapenem, Cephalosporin (4th), Fluroquinolone, Monobactam, Polymyxin, Rifabutin, TMP/SMX, Tetracycline | 1 (2·0) | 0 | 0 | 0 | 0 | 0 | 0 | 0 |
| Aminoglycoside, Carbapenem, Fluroquinolone, Macrolide | 0 | 0 | 0 | 1 (10·0) | 0 | 0 | 0 | 0 |
| Aminoglycoside, Carbapenem, Fluroquinolone, Monobactam, Penicillin | 1 (2·0) | 0 | 0 | 0 | 0 | 0 | 0 | 0 |
| Aminoglycoside, Carbapenem, Monobactam, Polymyxin | 0 | 0 | 0 | 0 | 0 | 0 | 1 (1·6) | 0 |
| Aminoglycoside, Carbapenem, Polymyxin, Tigecycline | 0 | 1 (1·1) | 0 | 0 | 0 | 0 | 0 | 0 |
| Aminoglycoside, Macrolide, Monobactam, Tigecycline | 0 | 0 | 0 | 0 | 0 | 0 | 1 (1·6) | 0 |
| Beta-lactamase, Carbapenem, Fluroquinolone, Macrolide, TMP/SMX | 0 | 1 (1·1) | 0 | 0 | 0 | 0 | 0 | 0 |
| Beta-lactamase, Carbapenem, Penicillin + beta-lactamase inhibitor, Polymyxin, Rifabutin, Tigecycline | 0 | 1 (1·1) | 0 | 0 | 0 | 0 | 0 | 0 |
| Beta-lactamase, Carbapenem, TMP/SMX, Tigecycline | 0 | 1 (1·1) | 0 | 0 | 0 | 0 | 0 | 0 |
| Beta-lactamase, Cephalosporin (4th), Macrolide, Polymyxin | 1 (2·0) | 0 | 0 | 0 | 0 | 0 | 0 | 0 |
| Beta-lactamase, Polymyxin, Rifabutin, Tigecycline | 0 | 1 (1·1) | 0 | 0 | 0 | 0 | 0 | 0 |
| Carbapenem, Polymyxin, TMP/SMX, Tigecycline | 0 | 0 | 0 | 1 (10·0) | 0 | 0 | 0 | 0 |
| AP = Acute Pyelonephritis; CRE = Carbapenem-Resistant Enterobacteriaceae; cUTI = Complicated Urinary Tract Infection; HABP = Hospital-acquired Bacterial Pneumonia; VABP = Ventilator-associated Bacterial Pneumonia  Cephalosporin (3rd) = 3rd Generation Cephalosporin; 4th Generation Cephalosporin; TMP/SMX = Trimethoprim-Sulfamethoxazole; Beta-lactam = Extended spectrum beta lactam; Beta-lactamase = Extended-spectrum beta-lactam (ureidopenicillin) + beta-lactamase inhibitor | | | | | | | | |

| **Supplemental Table D**.   Directed Antimicrobial Regimens Stratified by Site of Infection and Region of Origin (All Cases) | | | | | | | | |
| --- | --- | --- | --- | --- | --- | --- | --- | --- |
|  | **Bacteremia** | | **HABP** | | **VABP** | | **cUTI/AP** | |
| **Directed Antimicrobial Therapy** | **US (N=50) n (%)** | **EU (N=90) n (%)** | **US (N=11) n (%)** | **EU (N=10) n (%)** | **US (N=10) n (%)** | **EU (N=10) n (%)** | **US (N=61) n (%)** | **EU (N=14) n (%)** |
| **Monotherapy** | **14 (28·0)** | **7 (7·8)** | **4 (36·4)** | **1 (10·0)** | **1 ( 10·0)** | **1 (10·0)** | **34 (5·7)** | **2 (14·3)** |
| Fluroquinolone | 3 (6·0) | 0 | 3 (27·3) | 0 | 0 | 0 | 7 (11·5) | 0 |
| Polymyxin | 1 (2·0) | 4 (4·4) | 0 | 0 | 1 (10·0) | 1 (10·0) | 5 (8·2) | 0 |
| Aminoglycoside | 3 (6·0) | 2 (2·2) | 0 | 0 | 0 | 0 | 6 (9·8) | 0 |
| Carbapenem | 3 (6·0) | 0 | 0 | 0 | 0 | 0 | 3 (4·9) | 0 |
| Phosphonic | 0 | 0 | 0 | 0 | 0 | 0 | 5 (8·2) | 0 |
| TMP/SMX | 2 (4·0) | 0 | 0 | 0 | 0 | 0 | 2 (3·3) | 0 |
| Cephalosporin (3rd) | 0 | 0 | 0 | 1 (10·0) | 0 | 0 | 2 (3·3) | 0 |
| Tigecycline | 1 (2·0) | 1 (1·1) | 0 | 0 | 0 | 0 | 1 (1·6) | 0 |
| Beta-lactam | 0 | 0 | 0 | 0 | 0 | 0 | 0 | **2 (14·3)** |
| Beta-lactamase | 0 | 0 | 1 (9·1) | 0 | 0 | 0 | 1 (1·6) | 0 |
| Cephalosporin (4th) | 0 | 0 | 0 | 0 | 0 | 0 | 1 (1·6) | 0 |
| Monobactam | 1 (2·0) | 0 | 0 | 0 | 0 | 0 | 0 | 0 |
| Nitrofurantoins | 0 | 0 | 0 | 0 | 0 | 0 | 1 (1·6) | 0 |
| **Dual Therapy** | **10 (20·0)** | **29 (32·2)** | **1 (9·1)** | **2 (20·0)** | **3 (30·0)** | **2 (20·0)** | **5 (8·2)** | **3 (21.3)** |
| Carbapenem, Polymyxin | 1 (2·0) | 10 (11·1) | 1 (9·1) | 0 | 0 | 1 (10·0) | 0 | 0 |
| Aminoglycoside, Tigecycline | 2 (4·0) | 4 (4·4) | 0 | 2 (20·0) | 0 | 0 | 1 (1·6) | 0 |
| Polymyxin, Tigecycline | 3 (6·0) | 1 (1·1) | 0 | 0 | 1 (10·0) | 0 | 0 | 1 (7.1) |
| Aminoglycoside, Polymyxin | 0 | 3 (3·3) | 0 | 0 | 0 | 0 | 0 | 1 (7.1) |
| Aminoglycoside, Beta-lactam | 0 | 3 (3·3) | 0 | 0 | 0 | 0 | 0 | 0 |
| Aminoglycoside, Carbapenem | 0 | 2 (2·2) | 0 | 0 | 0 | 0 | 1 (1·6) | 0 |
| Carbapenem, Tigecycline | 0 | 2 (2·2) | 0 | 0 | 0 | 0 | 0 | 0 |
| TMP/SMX, Tigecycline | 1 (2·0) | 0 | 0 | 0 | 1 (10·0) | 0 | 0 | 0 |
| Aminoglycoside, Fluroquinolone | 0 | 1 (1·1) | 0 | 0 | 0 | 0 | 0 | 0 |
| Aminoglycoside, Phosphonic | 0 | 0 | 0 | 0 | 0 | 0 | 0 | 1 (7.1) |
| Beta-lactam, Polymyxin | 0 | 1 (1·1) | 0 | 0 | 0 | 0 | 0 | 0 |
| Beta-lactamase, Polymyxin | 0 | 0 | 0 | 0 | 0 | 1 (10·0) | 0 | 0 |
| Beta-lactamase, TMP/SMX | 1 (2·0) | 0 | 0 | 0 | 0 | 0 | 0 | 0 |
| Carbapenem, Cephalosporin (4th) | 0 | 0 | 0 | 0 | 1 (10·0) | 0 | 0 | 0 |
| Carbapenem, Nitrofurantoins | 0 | 0 | 0 | 0 | 0 | 0 | 1 (1·6) | 0 |
| Carbapenem, TMP/SMX | 0 | 1 (1·1) | 0 | 0 | 0 | 0 | 0 | 0 |
| Cephalosporin (4th), Tigecycline | 1 (2·0) | 0 | 0 | 0 | 0 | 0 | 0 | 0 |
| Fluroquinolone, Penicillin + beta-lactamase inhibitor | 1 (2·0) | 0 | 0 | 0 | 0 | 0 | 0 | 0 |
| Fluroquinolone, TMP/SMX | 0 | 0 | 0 | 0 | 0 | 0 | 1 (1·6) | 0 |
| Penicillin + beta-lactamase inhibitor, Polymyxin | 0 | 0 | 0 | 0 | 0 | 0 | 1 (1·6) | 0 |
| Phosphonic, Tigecycline | 0 | 1 (1·1) | 0 | 0 | 0 | 0 | 0 | 0 |
| **3-Drug Combinations** | **10 (20·0)** | **40 (44·4)** | **2 (18·2)** | **5 (50·0)** | **3 (30·0)** | **6 (60·0)** | **2 (3·3)** | **6 (42.9)** |
| Carbapenem, Polymyxin, Tigecycline | 3 (6·0) | 11 (12·2) | 0 | 2 (20·0) | 0 | 4 (40·0) | 0 | 2 (14·3) |
| Aminoglycoside, Carbapenem, Tigecycline | 2 (4·0) | 12 (13·3) | 0 | 0 | 0 | 0 | 1 (1·6) | 1 (7.1) |
| Aminoglycoside, Carbapenem, Polymyxin | 0 | 6 (6·7) | 2 (18·2) | 1 (10·0) | 0 | 2 (20·0) | 0 | 2 (14·3) |
| Aminoglycoside, Polymyxin, Tigecycline | 2 (4·0) | 3 (3·3) | 0 | 0 | 0 | 0 | 0 | 0 |
| Polymyxin, Rifabutin, Tigecycline | 0 | 2 (2·2) | 0 | 1 (10·0) | 0 | 0 | 0 | 0 |
| Aminoglycoside, Beta-lactam, Fluroquinolone | 0 | 2 (2·2) | 0 | 0 | 0 | 0 | 0 | 0 |
| Aminoglycoside, Cephalosporin (4th), Tigecycline | 1 (2·0) | 0 | 0 | 0 | 1 (10·0) | 0 | 0 | 0 |
| Carbapenem, Rifabutin, Tigecycline | 0 | 2 (2·2) | 0 | 0 | 0 | 0 | 0 | 0 |
| Aminoglycoside, Carbapenem, Phosphonic | 0 | 1 (1·1) | 0 | 0 | 0 | 0 | 0 | 0 |
| Aminoglycoside, Carbapenem, Rifabutin | 0 | 1 (1·1) | 0 | 0 | 0 | 0 | 0 | 0 |
| Aminoglycoside, Cephalosporin (4th), Fluroquinolone | 1 (2·0) | 0 | 0 | 0 | 0 | 0 | 0 | 0 |
| Aminoglycoside, Cephalosporin (4th), Polymyxin | 0 | 0 | 0 | 0 | 1 (10·0) | 0 | 0 | 0 |
| Carbapenem, Phosphonic, Rifabutin | 0 | 0 | 0 | 0 | 0 | 0 | 0 | 1 (7.1) |
| Cephalosporin (4th), Polymyxin, Tigecycline | 0 | 0 | 0 | 0 | 0 | 0 | 1 (1·6) | 0 |
| Fluroquinolone, Macrolide, Polymyxin | 1 (2·0) | 0 | 0 | 0 | 0 | 0 | 0 | 0 |
| Fluroquinolone, Macrolide, TMP/SMX | 0 | 0 | 0 | 0 | 1 (10·0) | 0 | 0 | 0 |
| Polymyxin, TMP/SMX, Tigecycline | 0 | 0 | 0 | 1 (10·0) | 0 | 0 | 0 | 0 |
| **4 and more Drug Combinations** | **7 (14·0)** | **8 (8·9)** | **1 (9·1)** | **1 (10·0)** | **1 (10·0)** | **1 (10·0)** | **1 (1·6)** | 1 (7.1) |
| Aminoglycoside, Beta-lactamase, Polymyxin, Tigecycline | 1 (2·0) | 0 | 0 | 0 | 1 (10·0) | 0 | 0 | 0 |
| Aminoglycoside, Carbapenem, Polymyxin, Tigecycline | 1 (2·0) | 1 (1·1) | 0 | 0 | 0 | 0 | 0 | 0 |
| Carbapenem, Polymyxin, Rifabutin, Tigecycline | 0 | 1 (1·1) | 0 | 0 | 0 | 1 (10·0) | 0 | 0 |
| Aminoglycoside, Carbapenem, Cephalosporin (3rd), Tigecycline | 1 (2·0) | 0 | 0 | 0 | 0 | 0 | 0 | 0 |
| Aminoglycoside, Carbapenem, Fluroquinolone, Macrolide, TMP/SMX, Tigecycline | 0 | 0 | 1 (9·1) | 0 | 0 | 0 | 0 | 0 |
| Aminoglycoside, Carbapenem, Fluroquinolone, Tigecycline | 1 (2·0) | 0 | 0 | 0 | 0 | 0 | 0 | 0 |
| Aminoglycoside, Carbapenem, Macrolide, Polymyxin | 0 | 0 | 0 | 1 (10·0) | 0 | 0 | 0 | 0 |
| Aminoglycoside, Carbapenem, Phosphonic, Polymyxin, Tigecycline | 0 | 0 | 0 | 0 | 0 | 0 | 0 | 1 (7.1) |
| Aminoglycoside, Carbapenem, Polymyxin, Rifabutin | 0 | 1 (1·1) | 0 | 0 | 0 | 0 | 0 | 0 |
| Aminoglycoside, Carbapenem, Polymyxin, Rifabutin, Tigecycline | 0 | 1 (1·1) | 0 | 0 | 0 | 0 | 0 | 0 |
| Aminoglycoside, Carbapenem, Polymyxin, TMP/SMX, Tigecycline | 0 | 1 (1·1) | 0 | 0 | 0 | 0 | 0 | 0 |
| Aminoglycoside, Cephalosporin (4th), Polymyxin, Tigecycline | 0 | 0 | 0 | 0 | 0 | 0 | 1 (1·6) | 0 |
| Aminoglycoside, Chloramphenicol, Rifabutin, Tigecycline | 1 (2·0) | 0 | 0 | 0 | 0 | 0 | 0 | 0 |
| Aminoglycoside, Fluroquinolone, Polymyxin, Tigecycline | 0 | 1 (1·1) | 0 | 0 | 0 | 0 | 0 | 0 |
| Aminoglycoside, Polymyxin, Rifabutin, Tigecycline | 0 | 1 (1·1) | 0 | 0 | 0 | 0 | 0 | 0 |
| Beta-lactamase, Cephalosporin (3rd), Cephalosporin (4th), Fluroquinolone | 1 (2·0) | 0 | 0 | 0 | 0 | 0 | 0 | 0 |
| Carbapenem, Phosphonic, Polymyxin, Rifabutin, Tigecycline | 0 | 1 (1·1) | 0 | 0 | 0 | 0 | 0 | 0 |
| Carbapenem, Polymyxin, Tetracycline, Tigecycline | 1 (2·0) | 0 | 0 | 0 | 0 | 0 | 0 | 0 |
| AP = Acute Pyelonephritis; CRE = Carbapenem-Resistant Enterobacteriaceae; cUTI = Complicated Urinary Tract Infection; HABP = Hospital-acquired Bacterial Pneumonia; VABP = Ventilator-associated Bacterial Pneumonia  Cephalosporin (3rd) = 3rd Generation Cephalosporin; 4th Generation Cephalosporin; TMP/SMX = Trimethoprim-Sulfamethoxazole; Beta-lactam = Extended spectrum beta lactam; Beta-lactamase = Extended-spectrum beta-lactam (ureidopenicillin) + beta-lactamase inhibitor | | | | | | | | |

**Supplemental Table E.** Multivariate Analysis of Factors Associated with 28-Day Mortality Among All Infection Types

| **Category** | **Nonsurvivors**  **(N=72)** | **Survivors**  **(N=157)** | **Bivariate OR**  **(95% CI)** | **Multivariate OR**  **(95% CI)** |
| --- | --- | --- | --- | --- |
| Gender, male, n (%) | 41 (56.9%) | 96 (61.1%) |  |  |
| Prior duration of hospitalization^1^ > 13 days, n (%) | 43 (59.7%) | 71 (45.2%) | 1.8 (1.0-3.2) | 1.9 (1.0 -3.5) |
| Immunocompromised condition^2^, n (%) | 21 (29.2%) | 41 (26.1%) |  |  |
| Comorbidities, n (%) |  |  |  |  |
| Diabetes mellitus, n (%) | 27 (37.5%) | 39 (24.8%) |  |  |
| Heart failure, n (%) | 23 (31.9%) | 25 (15.9%) | 2.5 (1.3-4.8) | 2.4 (1.20-4.8) |
| Chronic renal insufficiency^3^, n (%) | 28 (38.9%) | 49 (31.2%) |  |  |
| Requirement for dialysis, n (%) | 21 (29.2%) | 26 (16.6%) | 2.1 (1.1-4.0) | NS^6^ |
| Solid tumor, n (%) | 19 (26.4%) | 30 (19.1%) |  |  |
| Hematologic malignancy, n (%) | 12 (1.7%) | 20 (12.7%) |  |  |
| Inadequate empiric treatment^4^, n (%) | 48 (66.7%) | 106 (67.5%) |  |  |
| Presentation with severe sepsis^5^, n (%) | 37 (51.4%) | 40 (25.5%) | 3.1 (1.7-5.6) | 2.7 (1.5-5.0) |

^1^Duration of hospitalization prior to index CRE infection.

^2^ Immunocompromised condition included hematologic malignancy, prior bone marrow transplant, or received immunosuppressive therapy, such

as cancer chemotherapy, anti-rejection medications for transplantation, or long term (≥2 weeks) use of systemic steroids.

^3^Chronic renal insufficiency was defined as moderate-to-severe renal disease.

^4^ Empiric therapy without in vitro activity was defined as empiric antimicrobial therapy that did not contain at least one agent with in vitro activity

against the index CRE pathogen according to microbiologic data entered.

^5^ Severe sepsis was defined as infection associated with any of the following: hypotension (SBP < 90mmHg or a decrease in SBP of > 40mmHg from

baseline [if known] unresponsive to fluid challenge), hypothermia (core temperature <35.6°C or <96.1°F), or disseminated intravascular coagulation

(DIC) as evidenced by prothrombin time or partial thromboplastin time 2x the upper limit of normal or platelets less than 50% of the lower limit of normal.^13^

^6^NS Denotes not statistically significant

**Supplemental Table F.** Summary of Clinical Cure and 28-Day Mortality Rates According to Combination versus Monotherapy

for Empiric and Directed Therapy (2 Definitions Used) – All CRE Cases

|  | **Definition 1^1^:** | | |
| --- | --- | --- | --- |
| **Empiric Therapy** | **Combination Therapy**  **(N=77)** | **Monotherapy**  **(N=148)** | **Odds Ratio (95% CI)** |
| Number (%) with Clinical Cure (PI-ascertained) | 37 (48.1%) | 89 (60.1%) | 0.6 (0.3-1.0) |
| 28-day mortality, n (%) | 28 (36.4%) | 38 (25.7%) | 1.6 (0.9-3.0) |
|  | **Definition 2^2^:** | | |
| **Empiric Therapy** | **Combination Therapy**  **(N=13)** | **Monotherapy**  **(N=70)** | **Odds Ratio (95% CI)** |
| Number (%) with Clinical Cure (PI-ascertained) | 8 (61.5%) | 34 (48.6%) | 1.5 (0.5-5.1) |
| 28-day mortality, n (%) | 4 (30.8%) | 20 (28.6%) | 0.9 (0.2-3.9) |
|  | **Definition 1^1^:** | | |
| **Directed Therapy** | **Combination Therapy**  **(N=134)** | **Monotherapy**  **(N=80)** | **Odds Ratio (95% CI)** |
| Number (%) with Clinical Cure (PI-ascertained) | 82 (61.2%) | 51 (63.8%) | 0.8 (0.5-1.5) |
| 28-day mortality, n (%) | 35 (26.1%) | 18 (22.5%) | 1.2 (0.6-2.3) |
|  | **Definition 2^2^:** | | |
| **Directed Therapy** | **Combination Therapy**  **(N=53)** | **Monotherapy**  **(N=115)** | **Odds Ratio (95% CI)** |
| Number (%) with Clinical Cure (PI-ascertained) | 37 (69.8%) | 66 (57.4%) | 1.6 (0. 8-3.2) |
| 28-day mortality, n (%) | 11 (20.8%) | 31 (27.0%) | 0.6 (0.3-1.4) |

^1^ Combination therapy was defined as two or more agents with gram negative therapy used in combination (>3 days).

^2^ Combination therapy was defined as two or more agents with in vitro activity against the index CRE pathogen used in combination (>3 days).

**Supplemental Table G.** Outcomes of Infection due to Carbapenem-Resistant Enterobacteriaceae by Region.

|  | **Number (%) of Patients** | |  |  |
| --- | --- | --- | --- | --- |
|  | **Region** | |  |  |
| **Outcome** | **Europe**  **(n= 125)** | **US**  **(n= 132)** | **p-value** | **OR/Diff***  **(95% CI)** |
| **Duration of hospitalization for IV antibiotics (mean+SD)** | **16.3 (16.1)** | **12.0 (14.5)** | **0.03** | **-** |
| Duration of ICU stay (mean+SD) | 6.8 (12.1) | 9.1 (16.8) | NS | - |
| Number (%) with clinical cure (PI-ascertained)^1^ | 71 (59.2%) | 75 (58.6%) | - | NS |
| **28 day mortality (%)^2^** | **43 (38.4%)** | **29 (22.0%)** | **-** | **1.9 (1.1 to 3.4)** |

*For continuous endpoints, difference in LS means between Europe and US and its associated 95% confidence interval are presented; for categorical endpoints, odds ratio of Europe relative to US and its associated 95% CI are presented.

^1^Eight patients (4 from Europe, 4 from US) with missing data were excluded from analysis.

^2^Twenty-seven patients (12 for Europe, 15 for US) with missing 28-day mortality information were excluded from analysis.

**Supplemental Table H.** Outcomes of cUTI and Bacteremia due to Carbapenemase-Resistant Enterobacteriaceae by Region.

|  | | | | | | |
| --- | --- | --- | --- | --- | --- | --- |
|  | **cUTI** | | | **Bacteremia** | | |
| **Outcome** | **Europe (n=14)** | **U.S.**  **(n=61)** | **All**  **(n=75)** | **Europe (n=90)** | **U.S.**  **(n=50)** | **All**  **(n=140)** |
| Number (%) with clinical cure (PI-ascertained) | 11 (79%) | 43 (73%)^1^ | 54 (74%) | 52 (61%)^3^ | 22 (44%) | 74 (54%) |
| Number (%) with microbiological cure | 11 (79%) | 27 (51%)^2^ | 38 (57%) | 51 (65%)^4^ | 27 (54%) | 78 (61%) |
| Number (%) alive at Day 28^5^ | 7 (58%) | 44 (83.7%) | 51 (79%) | 50 (68%) | 30 (64%) | 80 (66%) |
| 28-day Mortality^5^ | 5 (42%) | 8 (15%)^6^ | 13 (20%) | 24 (32%) | 17 (36%) | 41 (34%) |

^1^ Does not include 2 patients with cUTI from US with missing clinical cure data;

^2^ Does not include 8 patients with cUTI from US with missing microbiological cure data;

^3^ Does not include 4 patients with bacteremia from Europe with missing clinical cure data

^4^ Does not include 11 patients with bacteremia from Europe with missing microbiological cure data

^5^ Does not include patients for whom mortality status on Day 28 was missing: 2 cUTI/AP from Europe, 9 cUTI/AP from U.S., 10 bacteremia from Europe, 3 bacteremia U.S.

^6^ Not statistically significant (OR 3.93; 95% CI 0.99-15.50)

**Supplemental Table I.** Nonsusceptible rates to agents commonly used to treat infections due to CRE pathogens according to region of origin –

US versus Europe.

|  |  | **Number (%) of Isolates** | | |  |  |
| --- | --- | --- | --- | --- | --- | --- |
| **cUTI** | **Susceptibility** | **US** | **Europe** | **All** | ***P*-Value** | **OR (% CI)** |
| Amikacin | Susceptible | 42 (82.4%) | 4 (40.0%) | 46 (75.4%) | 0.01 | 7.0 (1.63, 30.0) |
|  | Nonsusceptible | 9 (17.6%) | 6 (60.0%) | 15 (24.6%) |  |  |
| Tigecycline | Susceptible | 10 (66.7%) | 6 (54.5%) | 16 (61.5%) | 0.53 | 1.67 (0.34, 8.26) |
|  | Nonsusceptible | 5 (33.3%) | 5 (45.5%) | 10 (38.5%) |  |  |
| Gentamicin | Susceptible | 35 (57.4%) | 10 (62.5%) | 45 (58.4%) | 0.71 | 0.81 (0.26, 2.51) |
|  | Nonsusceptible | 26 (42.6%) | 6 (37.5%) | 32 (41.6%) |  |  |
| Tobramycin | Susceptible | 16 (27.1%) | 0 | 16 (26.2%) | - |  |
|  | Nonsusceptible | 43 (72.9%) | 2 (100.0%) | 45 (73.8%) |  |  |
| Colistin/Polymyxin B | Susceptible | 18 (85.7%) | 11 (73.3%) | 29 (80.6%) | 0.36 | 2.18 (0.41, 11.64) |
|  | Nonsusceptible | 3 (14.3%) | 4 (26.7%) | 7 (19.4%) |  |  |
| Ciprofloxacin | Susceptible | 9 (20.9%) | 0 | 9 (16.1%) | - |  |
|  | Nonsusceptible | 34 (79.1%) | 13 (100.0%) | 47 (83.9%) |  |  |
| **Bacteremia** |  |  |  |  |  |  |
| Amikacin | Susceptible | 22 (55.0%) | 14 (16.3%) | 36 (28.6%) | <0.001 | 6.286 (2.67, 14.65) |
|  | Nonsusceptible | 18 (45.0%) | 72 (83.7%) | 90 (71.4%) |  |  |
| Tigecycline | Susceptible | 25 (78.1%) | 42 (53.8%) | 67 (60.9%) | 0.02 | 3.06 (1.18, 7.91) |
|  | Nonsusceptible | 7 (21.9%) | 36 (46.2%) | 43 (39.1%) |  |  |
| Gentamycin | Susceptible | 20 (40.0%) | 54 (60.7%) | 74 (53.2%) | 0.02 | 0.43 (0.21, 0.887) |
|  | Nonsusceptible | 30 (60.0%) | 35 (39.3%) | 65 (46.8%) |  |  |
| Tobramycin | Susceptible | 2 (5.6%) | 5 (20.8%) | 7 (11.7%) | 0.09 | 0.22 (0.04, 1.275) |
|  | Nonsusceptible | 34 (94.4%) | 19 (79.2%) | 53 (88.3%) |  |  |
| Colistin/Polymyxin B | Susceptible | 21 (87.5%) | 57 (66.3%) | 78 (70.9%) | 0.05 | 3.56 (0.98, 12.93) |
|  | Nonsusceptible | 3 (12.5%) | 29 (33.7%) | 32 (29.1%) |  |  |
| Ciprofloxacin | Susceptible | 8 (19.5%) | 3 (3.9%) | 11 (9.3%) | 0.01 | 5.98 (1.49, 23.98) |
|  | Nonsusceptible | 33 (80.5%) | 74 (96.1%) | 107 (90.7%) |  |  |
| **All Indications** |  |  |  |  |  |  |
| Amikacin | Susceptible | 75 (69.4%) | 19 (16.5%) | 94 (42.2%) | <0.001 | 11.48 (6.05, 21.78) |
|  | Nonsusceptible | 33 (30.6%) | 96 (83.5%) | 129 (57.8%) |  |  |
| Tigecycline | Susceptible | 43 (78.2%) | 59 (55.1%) | 102 (63.0%) | 0.01 | 2.92 (1.38, 6.14) |
|  | Nonsusceptible | 12 (21.8%) | 48 (44.9%) | 60 (37.0%) |  |  |
| Gentamycin | Susceptible | 70 (52.6%) | 77 (61.6%) | 147 (57.0%) | 0.15 | 0.69 (0.42, 1.14) |
|  | Nonsusceptible | 63 (47.4%) | 48 (38.4%) | 111 (43.0%) |  |  |
| Tobramycin | Susceptible | 21 (19.1%) | 5 (16.1%) | 26 (18.4%) | 0.71 | 1.227 (0.42, 3.57) |
|  | Nonsusceptible | 89 (80.9%) | 26 (83.9%) | 115 (81.6%) |  |  |
| Colistin/Polymyxin B | Susceptible | 46 (86.8%) | 84 (69.4%) | 130 (74.7%) | 0.02 | 2.894 (1.20, 7.01) |
